# Core Gut Bacteria Analysis of Healthy Mice

**DOI:** 10.3389/fmicb.2019.00887

**Published:** 2019-04-24

**Authors:** Jingjing Wang, Tao Lang, Jian Shen, Juanjuan Dai, Ling Tian, Xingpeng Wang

**Affiliations:** ^1^Shanghai Key Laboratory for Pancreatic Diseases, Institute of Translational Medicine, Shanghai General Hospital, Shanghai Jiao Tong University School of Medicine, Shanghai, China; ^2^Ministry of Education Key Laboratory for Systems Biomedicine, Shanghai Centre for Systems Biomedicine, Shanghai Jiao Tong University, Shanghai, China

**Keywords:** core gut bacteria, ecological relationship, carbohydrate- and amino acid-metabolizing bacteria, immunomodulatory bacteria, probiotics, fecal microbiota transplantation

## Abstract

Previous studies revealed that there existed great individual variations of gut microbiota in mice, and the gut bacteria of mice were changed with the occurrence and development of diseases. To identify the core gut bacteria in healthy mice and explore their relationships with the host phenotypes would help to understand the underlying mechanisms. In this study, we identified 37 genus-level core bacteria from feces of 101 healthy mice with different ages, sexes, and mouse strains in three previous studies. They collectively represented nearly half of the total sequences, and predominantly included carbohydrate- and amino acids-metabolizing bacteria and immunomodulatory bacteria. Among them, *Anaerostipes* indwelt the gut of all healthy mice. Co-abundance analysis showed that these core genera were clustered into five groups (Group C1–C5), which were ecologically related. For example, the abundances of Group C2 including probiotics *Bifidobacterium* and *Lactobacillus* slightly positively correlated with those of Group C1. Principal component analysis (PCA) and multivariate analysis of variance test revealed that these core gut genera were distinguished with age and sex, and also associated with their health/disease state. Linear discriminant analysis effect size (LEfSe) method showed that bacteria in Group C1 and C2/C3 increased with the age in infancy and early adulthood, and were more abundant in female mice than in male ones. The metabolic syndrome (MS) induced by high fat diet (HFD) and accelerated postnatal growth would decrease Group C2 genera, whereas probiotics intervention would reverse HFD-induced reduction of Group C2. Spearman correlation analysis indicated that the principal components based on the abundance of the 37 core genera were significantly correlated with host characteristic parameters of MS. These results demonstrated that the 37 core genera in five co-abundance groups from healthy mice were related to host phenotypes. It was indicated that these prevalent gut bacterial genera could be representative of the healthy gut microbiome in gnotobiotic animal models, and might also be candidates of probiotics and fecal microbiota transplantation.

## Introduction

Gut microbiota is a highly complex ecosystem, with thousands of microbial species and great individual variations. It is known that gut microbiota modulates host immune system development and whole-body metabolism (Kau et al., 2011; Tremaroli and Backhed, 2012). The dysbiosis of gut microbiota will induce immunological and/or metabolic diseases, such as inflammatory bowel disease, colorectal cancer, and type 2 diabetes (Wong et al., 2017; Schirmer et al., 2018; Zhao et al., 2018). Besides the case–control gut microbiota study, the healthy core microbiota study focusing on the stable and permanent members of the community in normal and healthy populations has drawn considerable attention. It was reported that some bacterial phylogroups, including *Clostridium* XIVa, *Faecalibacterium, Ruminococcus, Bacteroides, Alistipes, Parabacteroides, Roseburia, Lachnospiraceae, Sporobacter, Dorea, Clostridium, Eubacterium, Collinsella, Coprococcus, Subdoligranulum, Streptococcus, Holdermania, Butyrivibrio, Anaerotruncus, Enterococcus, Blautia, Bifidobacterium, Anaerostipes, Lactobacillus, Phascolarctobacterium, Prevotella, Odoribacter, Veillonella, Eggerthella, Fusobacterium, Akkermansia, Escherichia, Bilophila, Oscillibacter, Desulfovibrio, Klebsiella*, or *Shigella*, accounted for 50–100% of the gut microbiota of the healthy individuals (Tap et al., 2009; Turnbaugh et al., 2009; Qin et al., 2010; Claesson et al., 2011; Sekelja et al., 2011; Xiao et al., 2015; Zhang J. et al., 2015; Cheng et al., 2016; Maier et al., 2018). No matter how the healthy core bacteria are defined and how different the identification results are in the population studies, it is certain that the prevalent and dominant core bacteria are pivotal to host gut homeostasis and health. So it is important to discover a comprehensive core microbiota profile for defining a healthy gut microbiota and guiding their intervention of host health.

Nevertheless, the ecological relationship of the members of core bacteria is not well understood. In a cohort study with 314 samples of healthy young Chinese, eight in nine core genera, including *Blautia, Clostridium, Ruminococcus, Faecalibacterium, Subdoligranulum, Roseburia, Coprococcus, Bacteroides*, were significantly positively correlated with each other, yet the core genus *Phascolarctobacterium* was negatively correlated with the other eight core genera (Zhang J. et al., 2015). In another study of dietary fibers treatment to genetic and simple obesity in children, species from *Eubacterium, Faecalibacterium, Roseburia, Clostridium, Alistipes, Subdoligranulum, Bacteroides, Parabacteroides, Flavonifractor* were positively correlated with each other, while *Bifidobacterium* including *B. pseudocatenulatum* and *B. breve*, showed negative correlation with other bacteria (Zhang C. et al., 2015). These results indicated that most of the prevalent bacteria were co-abundant and positively correlated. However, a healthy gut microbiota ecosystem should be full of cooperation and competition, which could keep the system stable (Zengler and Zaramela, 2018). Therefore, it is necessary to explore the ecology of core microbiota in healthy state including more bacteria without any selection pressures such as dietary intervention.

Thereinto, another crucial question is whether host phenotype is linked to the core gut bacteria or not. Some previous studies found that the core bacteria showed the ethnicity- and age-associated differences (Zhang J. et al., 2015; Cheng et al., 2016), yet some other ones did not involve the association between gut bacteria and host phenotype. More importantly, healthy gut microbiota are distinct from those of diseased hosts (Round and Mazmanian, 2009; Sekirov et al., 2010), so it should be made clear whether the discrepancy are mainly reflected in the stable core bacteria or the variable ones.

Investigation of the prevalent bacteria in mice feces would help to study human core gut bacteria. Firstly, mouse and human gut microbiota show similarity at the genus level even though the proportions are different (Xiao et al., 2015; Hugenholtz and de Vos, 2018). Secondly, the mouse gut microbiota is functionally similar to its human counterpart (Xiao et al., 2015). Meanwhile, genotype, diets, lifestyles and drugs, which are key factors to affect gut microbiota (Franks, 2011; Claesson et al., 2012; Conlon and Bird, 2014; Maier et al., 2018), can be strictly controlled in the mouse model so as to down out any outside noises and find out how the gut bacteria group work together. Moreover, many conclusions from mice studies are validated on subsequent clinical studies (Sivan et al., 2015; Routy et al., 2018).

Herein, we investigated the full profile of gut core bacteria at the genus level in healthy mouse. By using co-abundance analysis, we divided the gut core bacteria in groups, and further investigated the ecological relationships between groups. With multivariate statistical analysis, we identified the specific core bacteria associated with age, sex and health/disease state, and explored their role in host health. These results provided a comprehensive view on core gut bacteria and host health in mice. These prevalent core gut bacteria could be used as individual or cocktail candidates for probiotics and fecal microbiota transplantation.

## Materials and Methods

### Datasets

To search for the possible core bacteria in the mouse gut, we analyzed 16S rRNA gene sequences of mouse gut microbiota from three previously published datasets with accession numbers of SRP020353, SRP064646, and SRP076247 (Wang J. et al., 2015; Wang J. et al., 2016; Wang J.J. et al., 2016).

In the first dataset with the ID “PRO” (Wang J. et al., 2015), we collected 301568 fecal bacteria 16S rRNA V3 region sequences from 80 fecal samples of mice with or without metabolic syndrome (MS) and probiotics intervention (Table 1 and Supplementary Table S1). Briefly, 40 male specific pathogen-free (SPF) C57BL/6J mice at the age of 12 weeks were divided into five groups with eight mice a group. One group was fed on normal chow diet (NC), as the control group. The other four groups were fed on high fat diet (HFD) for 12 weeks to induce MS. Among them, three HFD groups were treated by three probiotics strains, respectively. At the end of 12 weeks, we examined the body weight, measured the fasting blood glucose and fasting insulin, carried out the area under the curve (AUC) of oral glucose tolerance tests (OGTT), as well as tested serum adiponectin, serum lipopolysaccharide-binding protein (LBP), and crown-like structures (CLS), cecal short-chain fatty acids (including acetate, propionate, butyrate, isobutyrate, valerate, isovalerate), and recorded the epididymal adipose tissue (eAT) adipocyte size. We also analyzed the mRNA levels of adiponectin, leptin, tumor necrosis factor-α (TNF-α), matrix metalloproteinase-12 (MMP-12), monocyte chemotactic protein-1 (MCP-1), CD11c in eAT, TNF-α in liver and jejunum. These host parameters were also used in our study.

**Table 1:** The fecal samples of healthy and disease mice from three datasets used in this study.

Dataset ID	PRO	CUG	FMT
***N* (healthy)**	48	34	19
Subset 1	40 male C57BL/6J mice (12 weeks)	11 male ICR mice (4 weeks)	10 male C57BL/6J mice (8 weeks)
Subset 2	8 male C57BL/6J mice (24 weeks)	12 male ICR mice (12 weeks)	9 female C57BL/6J mice (8 weeks)
Subset 3	–	11 male ICR mice (24 weeks)	–
***N* (sick)**	32	24	–
Subset 1	8 HFD-induced MS mice	36 NBW+APG mice	–
		(including three time points)	
Subset 2	24 probiotics-treated MS mice	36 LBW+APG mice	–
	(including three probiotic groups)	(including three time points)	
**SRA accession number**	SRP020353	SRP064646	SRP076247
**References**	Wang J. et al., 2015	Wang J. et al., 2016	Wang J.J. et al., 2016

In the second dataset CUG (Wang J. et al., 2016), we got 432428 fecal bacteria 16S rRNA V3 region sequences from 106 fecal samples of male SPF ICR mice at three time points (4, 12, and 24 weeks of age) (Table 1 and Supplementary Table S1). Three groups were divided in this dataset, and each group contained 12 mice. Besides of the control group, accelerated postnatal growth (APG) groups with normal birth weight (NBW+APG) and low birth weight (LBW+APG) were distinguished. The two APG groups (NBW+APG and LBW+APG) developed MS at the age of 4 weeks. The physiological index data of these grouped mice was also analyzed in this study, including body weight, weight of eAT, perirenal adipose tissue (pAT), subcutaneous adipose tissue (sAT), fasting blood glucose, fasting insulin, and cecal short-chain fatty acids levels.

The third dataset FMT (Wang J.J. et al., 2016) included 698564 fecal bacteria V3–V4 region sequences from 19 fecal samples of gnotobiotic mice (Table 1 and Supplementary Table S1). Ten male and 9 female germ-free C57BL/6J mice at the age of 7 weeks were involved. These mice were inoculated gut microbiota from a healthy man 1 week before fecal sample collection.

Taken together, there were 205 mice fecal samples in the three datasets (Supplementary Table S1). Among them, samples of 48, 34, and 19 healthy mice were in the dataset PRO, CUG, and FMT, respectively (totally 101 healthy mice) (Table 1).

### 16S rRNA Gene Sequence Processing

Raw data were clustered into operational taxonomic units (OTUs) as before (Wang J. et al., 2015; Wang J. et al., 2016; Wang J.J. et al., 2016), and showed in Supplementary Table S1. For each dataset, the OTUs which were identified as chimeras and singleton, and which contained fewer than 10 reads, and which present in fewer than 1% of samples were removed (Duvallet et al., 2017). The representative sequences of each OTU were subjected to RDP classifier (version 2.11) to determine the taxonomy (RDP 16S rRNA training set 16). The confidence threshold was 50% for the data of 454 pyrosequencing of V3 region, whereas the confidence cut off was 80% for the data of Illumina Miseq sequencing of V3–V4 region. The OTUs to genus was then collapsed, and the relative abundance of each genus in every fecal sample was calculated. The followed statistical analyses were all based on genus-level data to minimize batch effects.

### Co-abundance Analysis

The core genera were clustered into the core groups based on their abundance across all the 101 healthy mice samples using spearman correlation analysis. False discovery rate (FDR) was estimated using the Benjamini–Yekutieli method (Benjamini et al., 2001). It was considered significant when the absolute value of the correlation coefficient was more than 0.5 and the FDR value was less than 0.001.

### Alignment-Based Phylogenetic Analysis

To identify the phylogenetic relationships of the five core groups, the phylogenetic tree was reconstructed based on the sequences of the most abundant OTUs (named as OTU01 in the figures and tables) belonging to the 37 core gut bacteria in each dataset. Sequence alignment and phylogenetic analysis were performed using Mega 7. Then the neighbor-joining tree was constructed with 1,000 of bootstrap replications.

### Statistical Analysis

Principal component analysis (PCA) was performed to globally view the age-, sex-, and disease-associated gut microbiota structural segregation based on the Euclidean distances of the relative abundances of the 37 core genera. The statistical significance of PCA plots was assessed by multivariate analysis of variance (MANOVA) test in MATLAB R2011b (The MathWorks, Inc., Natick, MA, United States). The difference was considered significant when *p*-value of MANOVA was less than 0.05. Linear discriminant analysis effect size (LEfSe) were used to identify the differential core genera between different ages, sexes, and healthy status based on the relative abundance of 37 core genera. The OTUs were picked out when alpha value of the factorial Kruskal–Wallis test was <0.05 and the logarithmic LDA score was >2.0 (Segata et al., 2011). Spearman correlation analysis was used to evaluate the relationships between core microbiota based on the principle component scores of PCA and the individual host parameters of phenotype, gene expression, and metabolite. The correlations were considered significant when FDR was <0.1.

## Results

### Thirty-Seven Core Gut Bacteria Were Identified in Healthy Mice

We collected and re-analysis bacterial sequencing data from three previous studies to figure out the prevalent gut bacteria in the healthy mice. These data included 16S rRNA gene V3 or V3–V4 region sequences of fecal bacterium in mice with different ages, genders, strains, and healthy/disease state (Table 1 and Supplementary Table S1). We used OTUs clustered in the previous studies, and removed the OTUs potentially caused by sequencing mistakes (Supplementary Table S1). RDP classifier assigned the OTUs to 72, 72, and 48 genera in the database PRO, CUG, and FMT, respectively, and 101 genera in total (Supplementary Table S1 and Supplementary Figure S1). The 101 genera belonged to the following 10 phyla: Firmicutes (63 genera), Proteobacteria (12 genera), Bacteroidetes (11 genera), Actinobacteria (9 genera), Candidatus Saccharibacteria (1 genus), Deferribacteres (1 genus), Tenericutes (1 genus), Verrucomicrobia (1 genus), Fusobacteria (1 genus), Spirochaetes (1 genus) (Supplementary Figure S1). Among the 101 genera, 26 genera existed in all the three datasets, and 39 genera existed in two of them (Supplementary Figure S2).

**FIGURE 1:**
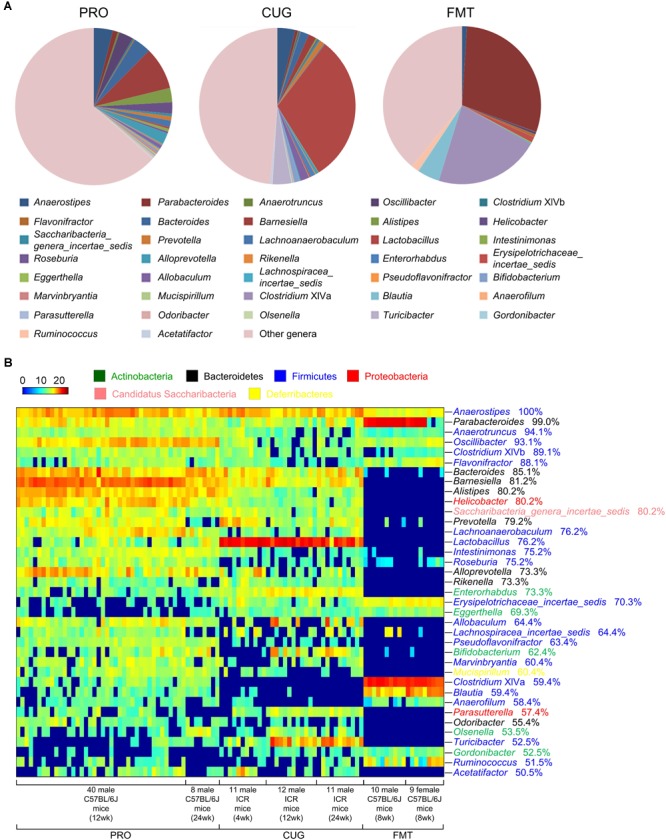
Thirty-seven core genera were identified in more than 50% of the feces of 101 healthy mice. **(A)** The proportion of each genus in the total sequences of each dataset. **(B)** The heatmap of the relative abundance (log_2_ transformed) of the 37 core genera in 101 healthy mice. Rows corresponded to the 37 core genera in the feces of 101 healthy mice. These genera were presented in order of prevalence (100–50.5%). The taxonomies of the genera were shown on the right, followed by the prevalence, and colored according to phylum. Columns represented 101 healthy mice samples in the three datasets. The 101 mice were grouped based on their sex, age, and strain.

We defined healthy core gut bacteria as those genera appeared in at least 50% of healthy mice samples regardless of their age, sex, and strain. In this study, there were 101 healthy mice samples in three datasets, which were from 40 male C57BL/6J mice of 12-week-old and 8 ones of 24-week-old in the database PRO, 11 male ICR mice of 4-week-old, 12 ones of 12-week-old and 11 ones of 24-week-old in the database CUG, 10 male C57BL/6J mice of 8-week-old, and 9 female ones of 8-week-old in the database FMT (Table 1). Across these 101 healthy mice, we identified 37 core genera. Among them, the most prevalence genera were the two, *Anaerostipes* (100% prevalence) and *Parabacteroides* (99.0%). The rest genera included *Anaerotruncus* (94.1%), *Oscillibacter* (93.1%), *Clostridium* XlVb (89.1%), *Flavonifractor* (88.1%), *Bacteroides* (85.1%), *Barnesiella* (81.2%), *Alistipes* (80.2%), *Helicobacter* (80.2%), *Saccharibacteria_genera_incertae_sedis* (80.2%), *Prevotella* (79.2%), *Lachnoanaerobaculum* (76.2%), *Lactobacillus* (76.2%), *Intestinimonas* (75.2%), *Roseburia* (75.2%), *Alloprevotella* (73.3%), *Rikenella* (73.3%), *Enterorhabdus* (73.3%), *Erysipelotrichaceae_incertae_sedis* (70.3%), *Eggerthella* (69.3%), *Allobaculum* (64.4%), *Lachnospiracea_incertae_sedis* (64.4%), *Pseudoflavonifractor* (63.4%), *Bifidobacterium* (62.4%), *Marvinbryantia* (60.4%), *Mucispirillum* (60.4%), *Clostridium* XIVa (59.4%), *Blautia* (59.4%), *Anaerofilum* (58.4%), *Parasutterella* (57.4%), *Odoribacter* (55.4%), *Olsenella* (53.5%), *Turicibacter* (52.5%), *Gordonibacter* (52.5%), *Ruminococcus* (51.5%), and *Acetatifactor* (50.5%) (Figure 1A,B and Supplementary Table S2).

The core genera were also highly abundant, ranking 36.60%, 51.49%, and 60.9% of the total sequences in dataset PRO, CUG, and FMT, respectively, and 46.77% on average (Figure 1A and Supplementary Table S2). Especially, it showed the highest proportion of gut microbiota (60.9%) in the dataset FMT even though the least core genera (22 core genera, 59.46%) were found, which might due to gavage transplantation of human gut microbiota into mice via oral gavage (Figure 1A and Supplementary Figure S3). Besides, there were 10 genera of the average relative abundances were over 1%, including *Lactobacillus* (10.51%), *Parabacteroides* (6.63%), *Barnesiella* (4.85%), *Anaerostipes* (3.29%), *Bacteroides* (2.35%), *Alistipes* (1.52%), *Oscillibacter* (1.51%), *Alloprevotella* (1.38%), *Turicibacter* (1.38%), and *Helicobacter* (1.21%), respectively (Figure 1B and Supplementary Table S2).

### The 37 Genera Were Divided Into Five Potential Core Groups

**FIGURE 2:**
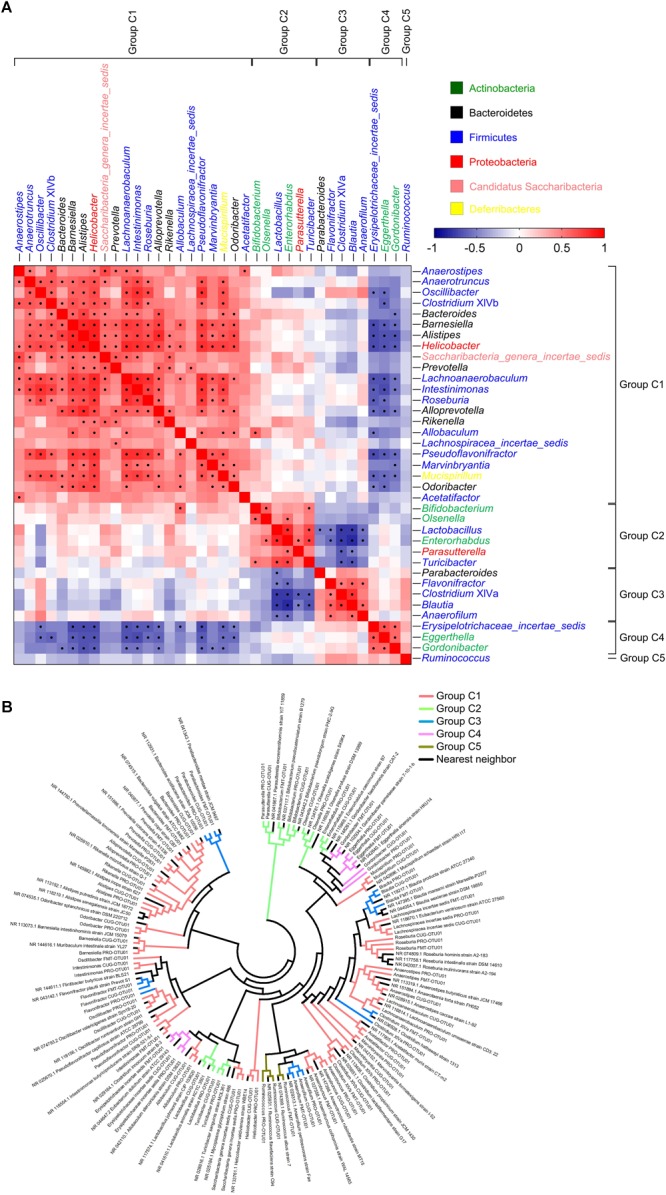
The 37 core genera were classified into five potential core groups by co-abundance analysis. **(A)** Interaction patterns of the 37 core genera across the 101 samples determined by Spearman correlation analysis. Rows and columns corresponded to the 37 core genera. These genera were with the names shown on the right and top, colored according to phylum, and grouped based on the co-abundance. Red and blue color denoted positive and negative association, respectively. The color intensity represented the degree of association. The black dots in the red/blue cells indicated the associations were significant (0.5 < | *r*| < 1, and false discovery rate < 0.001). In each core group, the genera were showed in order of prevalence. **(B)** Phylogenetic reconstruction of sequences to the 37 core genera. The most abundant OTUs (OTU01) of the 37 core genera in each dataset and their nearest neighbors were chosen to construct this neighbor-joining tree using Mega 7. The clades were colored according to the five core groups.

Spearman correlation analysis was further used to exhibit the relationships between any two co-abundance groups of the 37 core gut genera. The positively correlated co-abundance genera were considered as a core group. Accordingly, the 37 core genera were classified into 5 potential core groups (Figure 2A). Among them, Group C1 included 22 genera, including *Anaerostipes, Anaerotruncus, Oscillibacter, Clostridium* XlVb, *Bacteroides, Barnesiella, Alistipes, Helicobacter, Saccharibacteria_genera_incertae_sedis, Prevotella, Lachnoanaerobaculum, Intestinimonas, Roseburia, Alloprevotella, Rikenella, Allobaculum, Lachnospiracea_incertae_sedis, Pseudoflavonifractor, Marvinbryantia, Mucispirillum, Odoribacter* and *Acetatifactor*. Group C2 contained six genera, including *Bifidobacterium, Olsenella, Lactobacillus, Enterorhabdus, Parasutterella*, and *Turicibacter*. Group C3 covered five genera, including *Parabacteroides, Flavonifractor, Clostridium* XIVa, *Blautia*, and *Anaerofilum*. Group C4 comprised three genera, including *Erysipelotrichaceae _incertae_sedis, Eggerthella*, and *Gordonibacter*. Group C5 only constituted one genus, *Ruminococcus* (Figure 2A).

The five core groups were associated with each other (Figure 2A). Members in Group C1 was weakly positively correlated with those in Group C2. Especially, *Allobaculum* in Group C1 and *Bifidobacterium* in Group C2 were significantly positively correlated. Similarly, Group C3 showed positive association with Group C4 even if no statistical significance. Group C3 were significantly negatively correlated with Group C2, and Group C4 also showed significantly negative correlation with Group C1. However, Group C5 was the smallest group with only one genus, and was not correlated with any other groups.

To demonstrate the evolutionary relationship of the five core groups, we constructed the phylogenetic tree with the sequences of the most abundant OTUs belonging to the 37 core genera and their nearest neighbors (Figure 2B and Supplementary Tables S3, S4). Each OTU stayed together with its nearest neighbors (shown in Supplementary Table S4), and the OTUs from the three datasets in the same genus got together. Group C1 and Group C3 were widely distributed in the evolutionary tree, and most of them belonged to Firmicutes. Group C2 were clustered into two sub-groups: one belonged to Firmicutes, including *Lactobacillus* and *Turicibacter*, and the other included *Bifidobacterium, Olsenella, Enterorhabdus* (Actinobacteria), and *Parasutterella* (Proteobacteria), respectively. Group C4 gathered together. Group C5 were clustered with their nearest neighbors and *Anaerofilum* in Group C3.

### The 37 Genera Could Differentiate Gut Microbiota With Age and Sex

**FIGURE 3:**
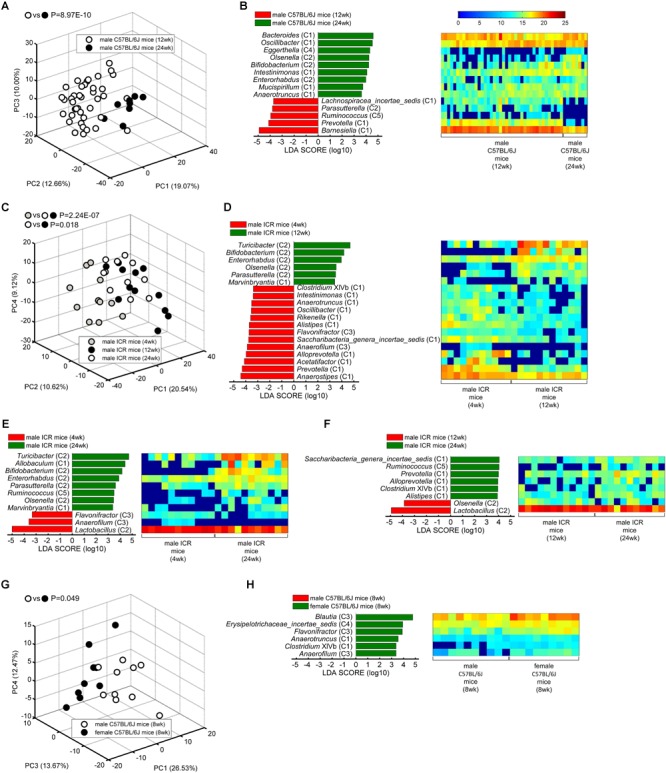
The core bacteria showed significant age- and sex-associated structural segregation. **(A,C,G)** PCA score plot based on the core genus abundance of healthy mice in dataset PRO **(A)**, CUG **(C)**, and FMT **(G)**. Each circular point represented the fecal core microbiota of a healthy mouse. *P*-values were calculated with MANOVA test, and shown on the top of PCA plots. **(B,D–F,H)** The different abundance of bacterial genus between the two groups were identified by LEfSe. It was significantly different when alpha value of the factorial Kruskal–Wallis test was <0.05 and the logarithmic LDA score was >2.0. The left histogram showed the LDA scores of genera differentially abundant between the two groups. The taxonomy was listed, followed by its core group. The right heatmap showed the relative abundance (log_2_ transformed) of the differential genera. *n* = 40 for male C57BL/6J mice (12 weeks), *n* = 8 for male C57BL/6J mice (24 weeks), *n* = 11 for male ICR mice (4 weeks), *n* = 12 for male ICR mice (12 weeks), *n* = 11 for male ICR mice (24 weeks), *n* = 10 for male C57BL/6J mice (8 weeks), and *n* = 9 for female C57BL/6J mice (8 weeks).

We wondered whether these healthy core genera could separate gut microbiota with different ages and sexes. So PCA was performed based on the abundance of the core genera, and MANOVA was used to confirm the statistically significant (Figure 3A,C,G). Further, linear discriminant analysis (LDA) effect size (LEfSe) was applied to identify the specific core bacteria which were separated in the two group mice (Figure 3B,D–F,H).

Principal component analysis showed that the core gut microbiota of 12-week-old male C57BL/6J mice were significantly different from those of 24-week-old ones (MANOVA, *p* = 8.97E-10) (Figure 3A). LEfSe LDA score bar and heatmap revealed that these abundance differences between the two group mice were increased in Group C1 (*Bacteroides, Oscillibacter, Intestinimonas, Mucispirillum, Anaerotruncus*), Group C2 (*Olsenella, Bifidobacterium, Enterorhabdus*), and Group C4 (*Eggerthella*), whilst decreased in Group C1 (*Barnesiella, Prevotella, Lachnospiracea_incertae_sedis*), Group C2 (*Parasutterella*), and Group C5 (*Ruminococcus*) (Figure 3B). Among the male ICR mice, PCA plots showed that the core gut bacteria in 4-week-old mice (infancy) were significantly separated with those of 12- and 24-week-old ones (adult) (MANOVA, *p* = 2.24E-07). As to the C57BL/6J mice, the samples of 12-week-old mice and 24-week-old ones were also significantly separated (MANOVA, *p* = 0.018) (Figure 3C). LEfSe results showed that the infant mice had more Group C1 and C3 than the adult mice, including *Anaerostipes* (C1), *Prevotella* (C1), *Acetatifactor* (C1), *Alloprevotella* (C1), *Saccharibacteria_genera_incertae_sedis* (C1), *Alistipes* (C1), *Rikenella* (C1), *Oscillibacter* (C1), *Anaerotruncus* (C1), *Intestinimonas* (C1), *Clostridium* XIVb (C1), *Anaerofilum* (C3), and *Flavonifractor* (C3), whilst less Group C2, including *Turicibacter, Bifidobacterium, Enterorhabdus, Olsenella, Parasutterella* (Figure 3D,E). In adult ICR mice, Group C1 (*Saccharibacteria_genera_incertae_sedis, Prevotella, Alloprevotella, Clostridium* XIVb, and *Alistipes*) and Group C5 (*Ruminococcus*) were increased, and Group C2 (*Olsenella, Lactobacillus*) were decreased, when the mice got older (Figure 3F). Taken together, these results indicated that the 37 core genera were age-associated.

The 37 core genera were also related to sex. PCA plots showed the core bacteria were significantly different between female and male C57BL/6J recipient mice transplanted by fecal microbiota from a healthy human (MANOVA, *p* = 0.049) (Figure 3G). LEfSe showed the female mice had more abundance of Group C1 (*Anaerotruncus, Clostridium* XIVb), Group C3 (*Blautia, Flavonifractor, Anaerofilum*) and Group C4 (*Erysipelotrichaceae_incertae_sedis*) genera than the male mice (Figure 3H).

### The 37 Core Genera Could Distinguish Gut Microbiota From Health and Disease State

We also wondered whether these 37 core genera were correlated with health/disease states. PCA was performed based on the abundance of the 37 core genera in healthy and sick mice (metabolic syndrome model, MS for short), and MANOVA was used to test the statistical significance of the differences between them. Spearman correlation analysis between the three principal components (PCs) of PCA was further carried out to determine the relationship of core bacteria and host disease state based on the core bacteria and host disease parameters in the dataset PRO or CUG. Then, different core genera between the two groups were picked out by LEfSe.

**FIGURE 4:**
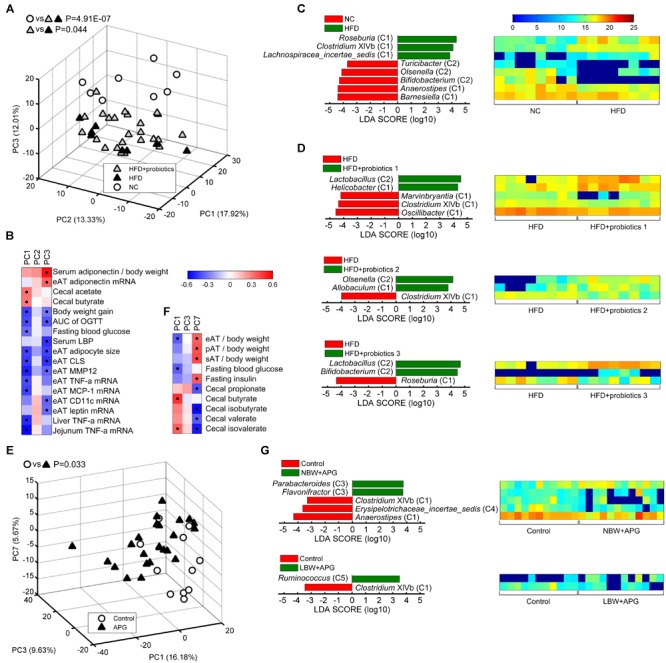
Core bacteria significantly distinguished the gut microbial structure of healthy and disease mice. **(A,E)** PCA score plot was presented based on the core genus abundance of healthy and disease mice in dataset PRO **(A)** and CUG **(E)**. Each circular or triangular point represented the fecal core microbiota of a healthy or disease mouse, respectively. *P*-values were calculated with MANOVA test, and shown on the top of PCA plots. **(B,F)** Spearman correlation between core microbiota represented by the three principal components (PCs) of PCA and host disease parameters of dataset PRO **(B)** and CUG **(F)**. Columns corresponded to the three PCs, and rows to the host disease parameters. Red and blue color denoted positive and negative correlations, respectively. The color intensity represented the correlation degree. The black dots in the red/blue cells indicated the correlations were significant (false discovery rate < 0.1). **(C,D,G)** The different abundance of bacterial genus between two groups was identified by LEfSe. It was significantly different when alpha value of the factorial Kruskal–Wallis test was <0.05 and the logarithmic LDA score was >2.0. The left histogram showed LDA scores of differentially abundant genera between the two groups. The taxonomy was listed, followed by its core group. The right heatmap showed the relative abundance (log_2_ transformed) of differential genera. *n* = 8 for NC, HFD, HFD+probiotics 1, HFD+probiotics 2, and HFD+probiotics three groups (*n* = 24 for HFD+probiotics group in total); *n* = 12 for Control, NBW+APG, LBW+APG groups (*n* = 24 for APG group in total).

In the dataset PRO, PCA plots showed HFD-induced MS group (shown in the figure as HFD) were significantly different from normal chow-fed control (shown in the figure as NC) (MANOVA, *p* = 4.91E-07), while probiotics treatment (shown in the figure as HFD+probiotics) could significantly reduce the HFD-induced harmful effects (MANOVA, *p* = 0.044) (Figure 4A). In addition, PC1 and PC3 was positively correlated with the protective parameters, including serum adiponectin/body weight, adiponectin mRNA in eAT, cecal acetate, and cecal butyrate, whilst negatively correlated with obesity and inflammation parameters, including body weight gain, AUC of OGTT, fasting blood glucose, serum LBP, eAT adipose size, eAT CLS, eAT MMP-12, eAT TNF-α mRNA, MCP-1 mRNA, CD11c mRNA, leptin mRNA, liver TNF-α mRNA, and jejunum TNF-α mRNA (Figure 4B). Then, LEfSe analysis indicated that HFD group had more Group C1 than NC (*Roseburia, Clostridium* XIVb, and *Lachnospiracea_incertae_sedis*), whilst less Group C2 (*Bifidobacterium, Olsenella*, and *Turicibacter*) and Group C1 (*Barnesiella* and *Anaerostipes*) (Figure 4C). However, probiotics treatment increased Group C2 including *Lactobacillus, Olsenella, Bifidobacterium*, and Group C1 including *Allobaculum*, whilst decreased Group C1 including *Roseburia* and *Clostridium* XIVb (Figure 4D). All these results suggested that these core genera were significantly related to HFD-induced MS and probiotics-mediated intervention.

In dataset CUG, PCA plots exhibited the APG group (shown in the figure as APG) was significantly separated from the control group (shown in the figure as Control) (MANOVA, *p* = 0.033) (Figure 4E). Spearman correlation heatmap showed PC1 of PCA was significantly positively correlated with cecal butyrate and cecal isovalerate, whilst negatively correlated with MS indices as eAT/body weight and fasting blood glucose. Meanwhile, PC7 showed significantly positive correlation with eAT/body weight, perirenal (p) AT/body weight, subcutaneous (s) AT/body weight and fasting insulin, whilst negative correlation with cecal propionate, isobutyrate, valerate, and isovalerate (Figure 4F). LEfSe results showed that APG raised the abundances of Group C3 (*Parabacteroides* and *Flavonifractor*) and Group C5 *Ruminococcus*, whilst suppressed the abundance of *Anaerostipes* (C1), *Erysipelotrichaceae_incertae_sedis* (C4), and *Clostridium* XIVb (C1) (Figure 4G). Together, the 37 core genera were also critical to the APG-induced MS.

## Discussion

Despite of the wide inter-individual structural variations, the composition of core gut bacteria and their roles in host phenotypes have not been completely solved yet. Hereinabove, we had exhibited a more comprehensive profile of core gut bacteria in healthy mouse model, excluding the influence of the diets, lifestyles and drugs. The core gut bacteria were divided into five core groups according to the co-abundant relationship, which might reflect their biological function to some extent. More importantly, the core gut bacteria were closely related to age, sex and health/disease state of the host, suggesting that the core bacteria might play a pivotal role in host gut homeostasis and health.

As mentioned above, the 37 core genera were identified across 101 healthy mice with different ages, sexes, and mouse stains at a prevalence of at least 50% in this study. Different from prior researches (Turnbaugh et al., 2009; Xiao et al., 2015; Zhang J. et al., 2015), we relaxed our restrictions to 50% prevalence to avoid losing the key taxa, which existed in different hosts but performed the same function. The 37 core bacteria presented 46.77% of total sequences on average, and could distinguish gut microbiota with age, sex, and health/disease state. These suggested that the 37 core genera could be the representatives of the whole gut microbiota and the indicators to the host phenotype. Notably, among the 37 core genera, *Anaerostipes* and *Parabacteroides* were the two most widespread bacteria, and *Anaerostipes* resided in the gut of all mice. It was reported that *Anaerostipes* species, such as *A. butyraticus*, and *A. caccae*, were butyrate-producing bacteria (Schwiertz et al., 2002; Eeckhaut et al., 2010), which was an important functional group in the gut of healthy hosts (Sekelja et al., 2011; Zhang J. et al., 2015; Cheng et al., 2016). *Parabacteroides* was at 99.0% prevalence. Previous studies showed *P. distasonis* and *P. goldsteinii* could induce gut or systemic inflammation (Awadel-Kariem et al., 2010; Kverka et al., 2011), and oral administration of *P. distasonis* antigens attenuated dextran sulfate sodium (DSS)-induced colitis in mice (Kverka et al., 2011). Considering the high prevalence (99%) and relatively high abundance (6.63% of relative abundance on average) of *Parabacteroides* in healthy mice, it is speculated that moderate *Parabacteroides* might stimulate the immune system and be essential to host health. Compared with previous studies, among the 37 core genera, 22 genera had been shown popular in the two previous studies, including *Alistipes, Bacteroides, Clostridium* XIVa, *Clostridium* XlVb, *Helicobacter, Lachnospiracea_incertae_sedis, Lactobacillus, Odoribacter, Parabacteroides, Rikenella, Roseburia, Turicibacter, Prevotella, Marvinbryantia, Ruminococcus, Blautia, Pseudoflavonifractor, Anaerotruncus, Oscillibacter, Eggerthella, Olsenella*, and *Bifidobacterium* (Benson et al., 2010; Xiao et al., 2015). Yet, 17 genera were reported in several human gut microbial compositional studies, such as *Anaerostipes, Parabacteroides, Anaerotruncus, Bacteroides, Alistipes, Prevotella, Lactobacillus, Roseburia, Eggerthella, Lachnospiracea_incertae_sedis, Bifidobacterium, Clostridium* XIVa, *Clostridium* XIVb, *Blautia, Odoribacter, Ruminococcus* and *Oscillibacter* (Tap et al., 2009; Turnbaugh et al., 2009; Qin et al., 2010; Claesson et al., 2011; Sekelja et al., 2011; Xiao et al., 2015; Zhang J. et al., 2015; Cheng et al., 2016; Maier et al., 2018). Therefore, there were commonalities between human and mouse core gut bacteria.

Now that these genera were indeed stable in the host gut, it is important to identify their function. In healthy mouse model, the natural ecological relationships were more visible, because the effect factors of gut microbiota, such as diets, lifestyles, and drugs, were controllable. Interestingly, these 37 core genera showed ecological networks and were divided into five core groups according to their co-abundant relationships. The core groups might represent the potential functional groups. Group C1, the largest group, included two predominant types of bacteria. One type of bacteria was butyrate-producing bacteria, such as *Anaerostipes, Anaerotruncus, Clostridium* XIVb, *Intestinimonas, Roseburia, Allobaculum, Lachnospiracea_incertae_sedis* (the nearest neighbor was *Eubacterium ventriosum*), *Pseudoflavonifractor*, and *Acetatifactor* (Barcenilla et al., 2000; Duncan et al., 2002; Schwiertz et al., 2002; Lau et al., 2006; Eeckhaut et al., 2010; Scott et al., 2011; Pfeiffer et al., 2012; Klaring et al., 2013; Machiels et al., 2014; Wang J. et al., 2015; Petri et al., 2017; Ueki et al., 2017). Butyrate is an essential bacterial metabolite from carbohydrate in the gut, as it is the preferred energy source for colon epithelial cells and maintains gut barrier functions (Peng et al., 2007). Meanwhile, butyrate has immunomodulatory and anti-inflammatory properties, and it attenuates inflammation by promoting differentiation and proliferation of regulatory T cells and inhibiting nuclear factor-κB activation (Furusawa et al., 2013; Kim et al., 2013; Smith et al., 2013). Administration of butyrate could alleviate gut inflammation (Singh et al., 2014). The other type of bacteria was immunity-activing or anti-infection or anti-tumor immunity-promoting bacteria, including *Bacteroides, Barnesiella, Alistipes, Helicobacter, Prevotella*, and *Mucispirillum* (Eaton et al., 2011; Ramanan et al., 2014; Bunker et al., 2015; Daillere et al., 2016; Kim and Kim, 2016; Moschen et al., 2016). These bacteria were critical to the immune systems of the host. If there were not these two types of bacteria in the gut, for example in germ-free mice, the immunity would be severely disordered (Hapfelmeier et al., 2010; Alam et al., 2011). Overall, these evidences indicated that Group C1 helped to shape and balance the immune system. Group C2 included carbohydrate-utilizing and lactate and/or acetate-producing bacteria, such as *Bifidobacterium, Olsenella*, and *Lactobacillus* (Fukuda et al., 2011; Matsuki et al., 2013; Li et al., 2015). *Bifidobacterium* and *Lactobacillus* were often used as probiotics to treat intestinal or systemic inflammation (Mohamadzadeh et al., 2011; Wang J. et al., 2015). Group C3 also contained some butyrate-producing bacteria, such as *Clostridium* XIVa and *Blautia* (Van den Abbeele et al., 2013; Takahashi et al., 2016), and some anti-inflammatory bacteria, like *Parabacteroides* and *Flavonifractor* (Kverka et al., 2011; Lee et al., 2018). It seemed that Group C3 had similar function with Group C1. This suggested that there were functional redundance in core gut bacteria. Functional redundance would keep system stable when it encountered perturbation. Group C5 only had one genus *Ruminococcus*, which contain bacteria conversing cellulose to acetate and hydrogen, such as *R. flavefaciens* and *R. albus* (Miller and Wolin, 1995; Koike and Kobayashi, 2001). Acetate and hydrogen were also showed to alleviate inflammation (Kajiya et al., 2009; Kim et al., 2013). Group C4 included two Actinobacteria *Eggerthella* and *Gordonibacter*, which were most phylogenetically closely to each other. Unlike the other core bacteria, these two bacteria metabolized amino acids rather than sugars (Lau et al., 2004; Woo et al., 2010). Altogether, members of the core microbiota in mice were principally carbohydrate- and amino acids-metabolizing bacteria and immunomodulatory bacteria. This was in accordance with previous functional studies on mice and human gut microbiota (Turnbaugh et al., 2009; Xiao et al., 2015).

In addition to intragroup co-abundance relationship, there were intergroup positive or negative correlation relationships. Group C1 and Group C2 were slightly positively correlated with each other. These interactions relied on nutrient and electron donor exchange (Zengler and Zaramela, 2018). For examples, polysaccharide, such as inulin, fructooligosaccharide or galactooligosaccharide, could be metabolized by *Bifidobacterium* or *Lactobacillus* in Group C2 into lactate and/or acetate. Then these products could be converted to butyrate by *Anaerostipes, Eubacterium* (the nearest neighbor of *Lachnospiracea_incertae_sedis*), *Allobaculum* or *Roseburia* in Group C1 (Schwiertz et al., 2002; Belenguer et al., 2006; Falony et al., 2006). That might be why *Bifidobacterium* or *Lactobacillus* could be chosen as probiotics to regulate the balance of gut microbiota. Group C3 was significantly correlated with Group C2 yet weakly negatively with Group C1. It might because *Clostridium* XIVa, *Blautia, Parabacteroides*, and *Flavonifractor* in Group C3 had the similar metabolic and anti-inflammatory functions with Group C1 and C2 (Kverka et al., 2011; Van den Abbeele et al., 2013; Takahashi et al., 2016; Lee et al., 2018). It also had been previously reported that *Blautia* (one genus in Group C3) were decreased by *Lactobacillus* (one genus in Group C2) (Wang R. et al., 2015). Bacteria in this group might be probiotics candidate and should be isolated, and their function should be tested in animal experiments. Group C4 was negatively correlated with Group C1, but the underlying mechanisms was unclear. Group C5 *Ruminococcus* was neutral with the other four groups, indicating that it might have more complete metabolic capacity and was independent to other members in the community. Together, these evidences revealed that there were social networks among the core gut bacteria in the healthy host. The network maintained the balance of host gut microenvironment. It is crucial to understand this complex network for developing strategies to reshape the gut microbial communities, such as making probiotic cocktail or standard fecal microbial transplantation.

We additionally demonstrated that age, sex and health/disease state were associated with the core gut bacteria. Unsupervised statistical method PCA showed that the 37 core genera were significantly separated according to different ages, sexes and health/disease state. Then, supervised multivariate statistical analysis was used to identify the specific genera in response to the corresponding variant. Compared with the infant mice, the adult mice had more abundant of probiotics-containing Group C2, which contrasted our mind that the infant gut resided more probiotics. Low abundant or lack of Group C2 in infancy might cause allergy, and supplement of Group C2 could prevent it (Kirjavainen et al., 2002; Kim et al., 2008). In the adult male C57BL/6J and ICR mice, Group C1 were increased in 24-week-old than in 12-week-old (*Bacteroides, Mucispirillum*, and *Anaerotruncus* for C57BL/6J mice; *Prevotella, Alistipes*, and *Clostridium* XIVb for ICR mice). Improving the immunoregulatory Group C1 and the probiotics-containing Group C2 with age in infancy and early adulthood would cause the core gut microbiota more and more mature and healthy (Zhang et al., 2012). Meanwhile, the female mice had more butyrate-producing and anti-inflammatory bacteria *Anaerotruncus, Clostridium* XIVb, *Blautia*, and *Flavonifractor* in Groups C1 and C3 than the male ones, suggesting that the females needed more anti-inflammatory bacteria to maintain intestinal health. This was in accordance with previous study that the female recipient mice transferred feces from a healthy man donor had more butyrate/acetate-producing bacteria and opportunistic pathogens (Wang J.J. et al., 2016). Moreover, in HFD-induced MS mice, butyrate-producing and immunity-promoting bacteria Group C1 (*Anaerostipes, Barnesiella*) and Group C2 (*Bifidobacterium, Olsenella, Turicibacter*) were decreased; while in APG group, Group C1 (*Anaerostipes, Clostridium* XIVb) were also reduced. These results indicated that the suffered MS was related to the reduction of Groups C1 and C2. In addition, the correlations between the core microbiota and the MS parameters were significantly. Together, the core microbiota identified in the present study distinguished gut microbiota not only with different ages and sexes in the healthy mice, but also with the health/disease state of mice. Thus, the core gut bacteria should be fully considered during disease treatment.

Although we identified a broad spectrum of mouse core gut bacteria covering 37 genera in this study, some bacteria that were dominant bacteria in human gut and related to the development or treatment of diseases were not in this list. As known, *Faecalibacterium* showed anti-inflammatory effects (Sokol et al., 2008), *Akkermansia* controlled obesity and diabetes (Plovier et al., 2017), and both of them were associated with anticancer immune responses in preclinical tumor models and cancer patients (Gopalakrishnan et al., 2018; Routy et al., 2018). However, these two important bacteria were not identified as the core in our present study. That might be due to *Faecalibacterium* inhabited in only 24 mice among the 101 healthy mice. Notably, 18 mice of these 24 ones were the mice transplanted with the healthy man feces, while only 6 mice were those with the native flora. These results suggested that *Faecalibacterium* were the dominant bacteria in human gut, but not in mouse gut. Besides, *Akkermansia* was only at a prevalence of 44.6% (45/101), and it might be identified as core gut bacteria if samples were enough. In addition to the protective bacteria *Faecalibacterium* and *Akkermansia*, the opportunistic pathogen *Escherichia/Shigella* (31/101) and *Desulfovibrio* (49/101) (Kotlowski et al., 2007; Dzierzewicz et al., 2010) were neither the core gut bacteria in this study. Similarly, *Desulfovibrio* might also be one of the core gut bacteria if the sample size enlarged. Among the 31 mice with *Escherichia/Shigella*, 14 mice were the ones with about 4,000 sequencing reads in one fecal sample (totally 19 mice), and 17 mice were with about 30,000 sequencing reads each sample (totally 82 mice). Thus, more core gut bacteria were identified if the sequencing depth was increasing.

In the present study, we clustered the data into genus to reduce batch effects in different sequencing sets (Duvallet et al., 2017). Another reason for the genus-level study was that the mouse gut microbiota was consistent with the humans’ ones at the genus level (Hugenholtz and de Vos, 2018). Even though we lost the sensitivity to detect species or strains, we found that the core gut bacteria, such as *Bifidobacterium, Lactobacillus, Clostridium*, and *Ruminococcus*, were in accordance with the metagenomics studies targeting bacterial strains (Qin et al., 2010; Xiao et al., 2015; Zhao et al., 2018). These indicated that the core study in genus level was preliminary but accurate.

## Conclusion

In conclusion, our results exhibited a relatively complete picture of core gut bacteria in healthy mice that they were mainly composed of carbohydrate- and amino acids-metabolizing bacteria and immunomodulatory bacteria, as well as their ecological relationships of symbiosis or functional redundancy. It was worth noting that the core bacteria resident in healthy gut was of fundamental importance for not only the healthy physiological characteristics, but also the chronic metabolic disease phenotypes. Our study focused on the gut microbiota of healthy host, and would be an important complement to the case–control study. Although this list of healthy core gut bacteria may need to supplement and correct by more researches, especially human studies, and extend from taxon core ones to functional core ones, it could be a treasury to screen the probiotics candidates or fecal microbial transplantation components for prevention and treatment of the diseases.

## Author Contributions

XW and JW designed the research. JS and JW provided the data sets. JW, TL, and JD analyzed the data. LT and JW discussed and interpreted the results. JW, TL, and LT wrote and revised the manuscript.

## Conflict of Interest Statement

The authors declare that the research was conducted in the absence of any commercial or financial relationships that could be construed as a potential conflict of interest.
