# Supplementary material for: Core Gut Bacteria Analysis of Healthy Mice
Source: Front Microbiol. 2019 Apr 24;10:887. doi: 10.3389/fmicb.2019.00887 (PMC6491893; doi:10.3389/fmicb.2019.00887)
Supplement: Supplementary file 1 [file Data_Sheet_1.PDF]

Core Gut microbiota analysis of feces in healthy mouse model

Jingjing Wang[1†], Tao Lang[1†], Jian Shen[2], Juanjuan Dai[1], Ling Tian[1*], Xingpeng Wang[1*]

Supplementary Material

Summary

The supplementary information includes three supplementary figures and four supplementary tables.

Supplementary figures

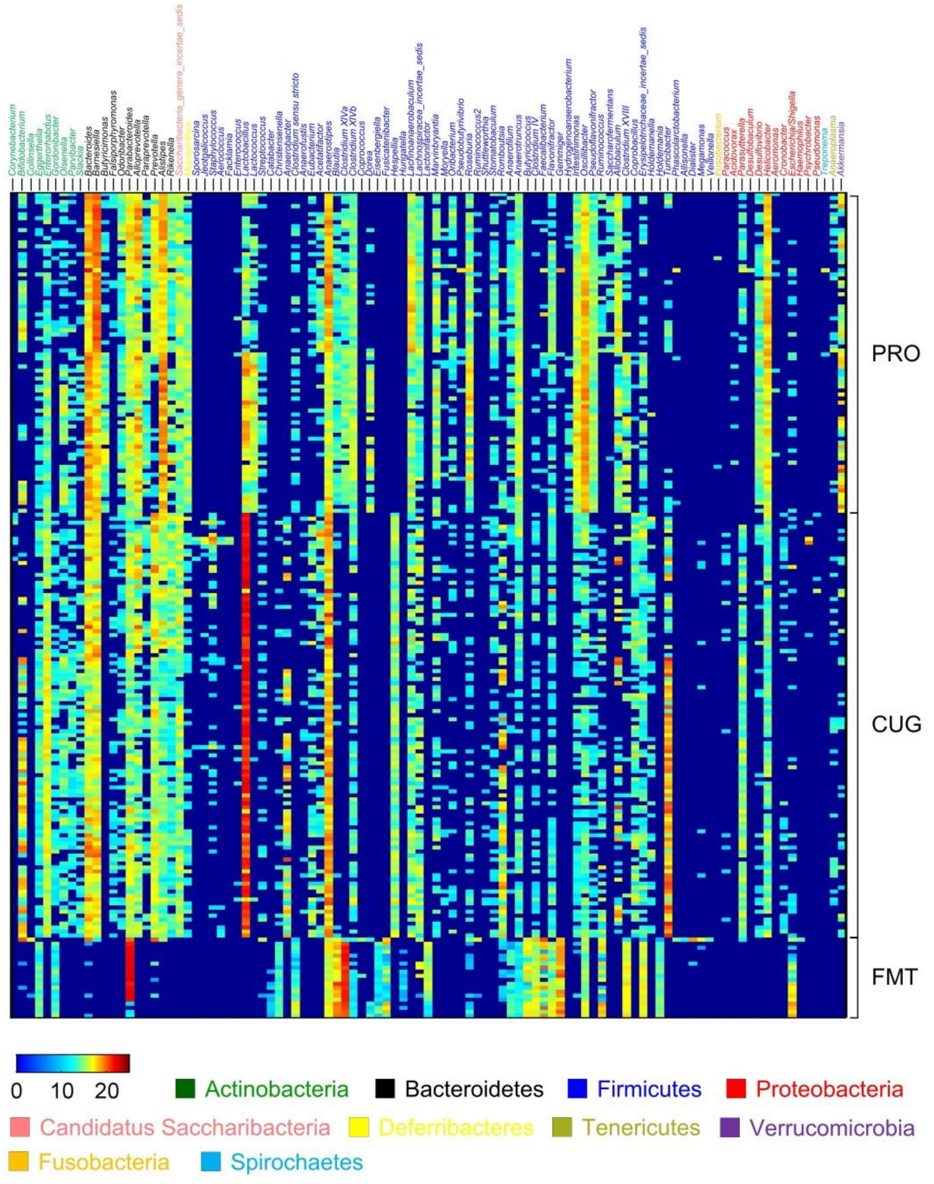

Supplementary Figure S1 Heatmap of the relative abundance of 101 genera in 205 mouse fecal samples. Data was log2 transformed. Rows represented 205 mice samples in the three datasets. Columns corresponded to 101 genera in all of these mice. These genera were ordered and colored according to phylum. The taxonomies of the genera were shown on the top.

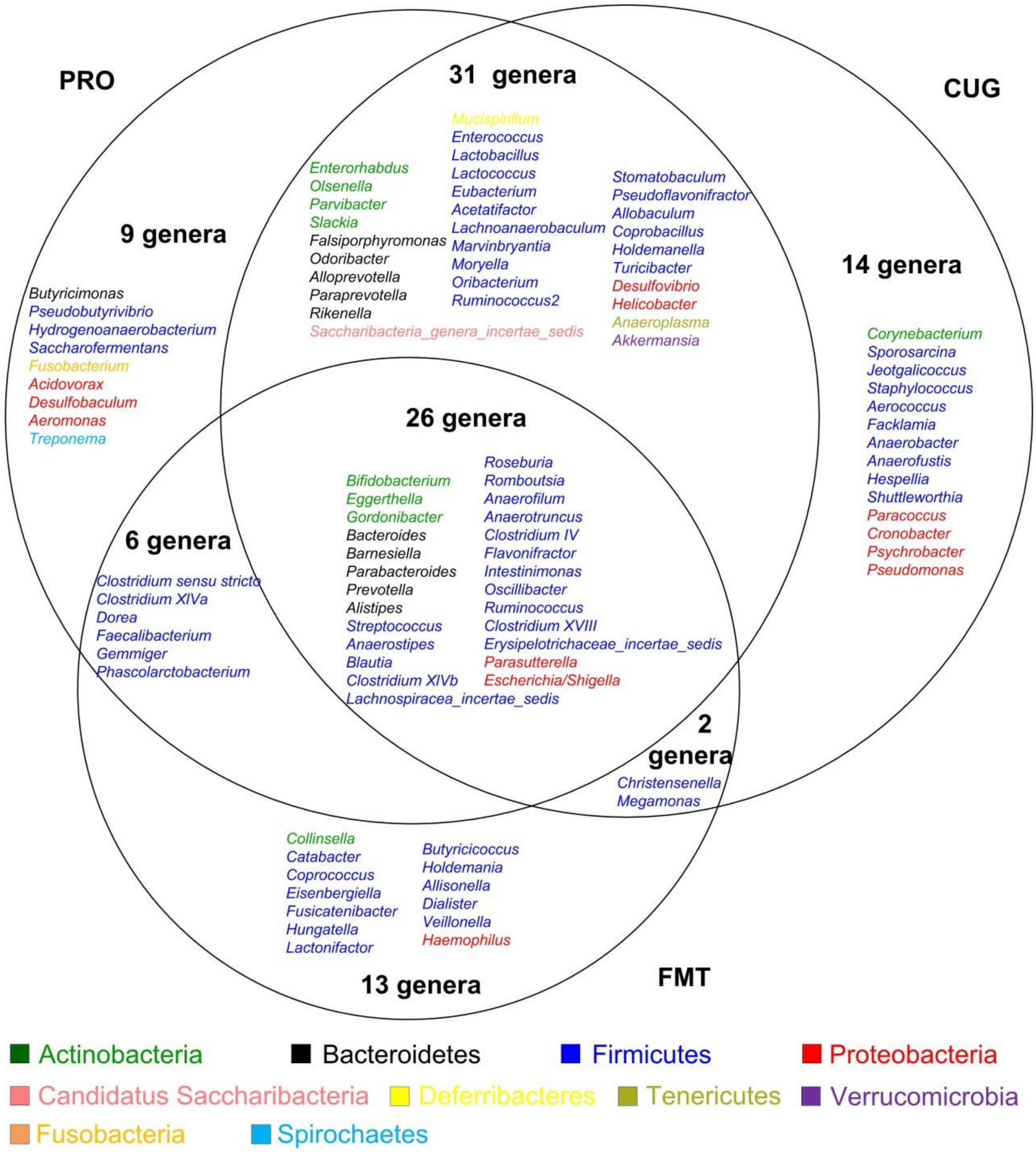

Supplementary Figure S2 Venn diagram of 101 genera in 205 mouse fecal samples. The genera' names were listed, and the colors of the genera were showed according to phylum.

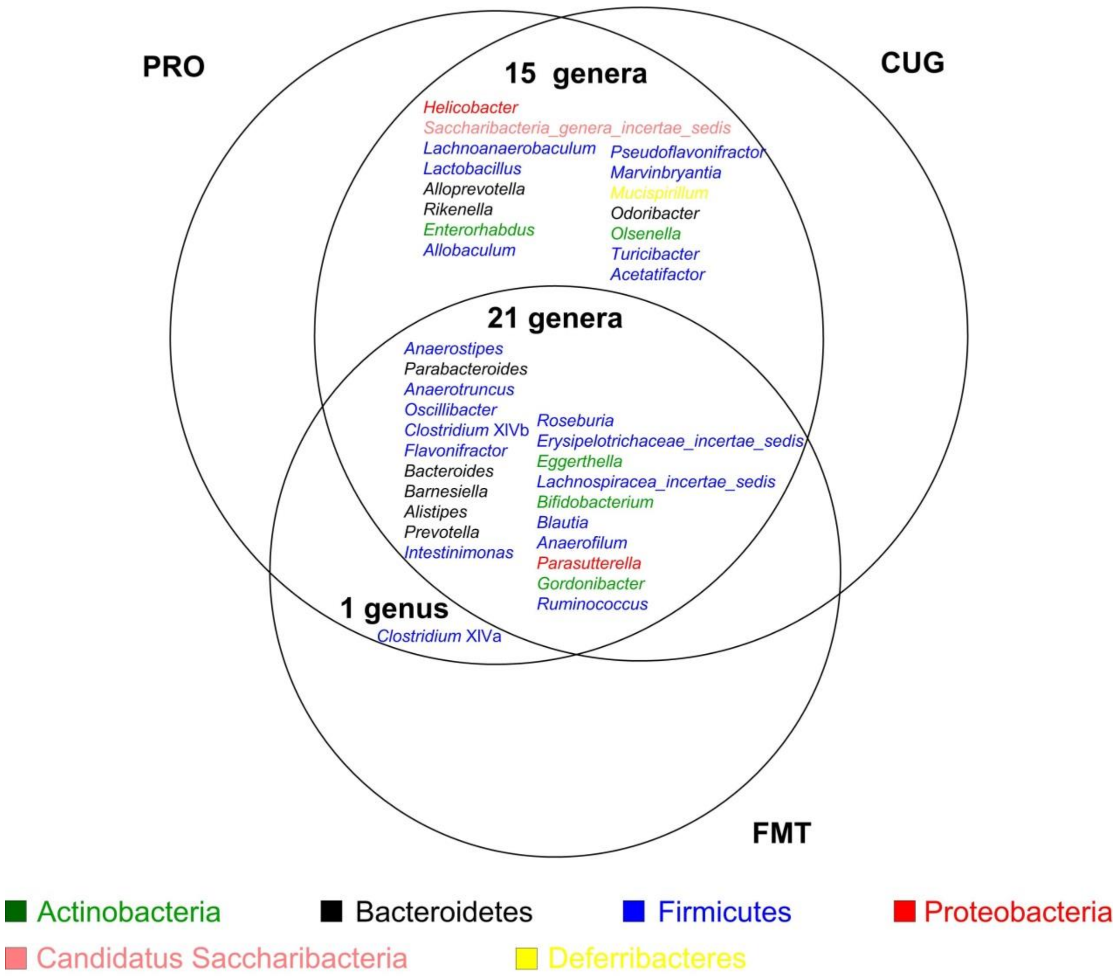

Supplementary Figure S3 Venn diagram of 37 genera in 101 healthy mouse fecal samples. The genera' names were listed, and the colors of the genera were showed according to phylum.

Supplementary tables

Supplementary Table S1 The sequence information of the three datasets

Dataset ID	PRO	CUG	FMT
**Sequencing platform**	454	454	Illumina Miseq
**Region of 16S rRNA gene**	V3	V3	V3-V4
**Total sample number**	80	106	19
**Raw sequences**	301568	432428	698564
**Average reads per sample**	3770±677	4079±992	33252±7415
**OTU number (similarity)**	2794 (98%)	987(98%)	2937 (97%)
**OTU number after removing chimeras and singleton**	1665	666	554
**OTU number after removing OTUs with <10 reads**	618	393	248
**OTU number after removing OTUs present in <1% of the samples**	618	392	248
**RDP bootstrap cut off**	50%	50%	80%
**Genus number**	72	72	48

Data were shown as means ±SEM.

Supplementary Table S2 The 37 healthy core genera identified in the feces of 101 healthy mice

Genus	Phylum	Prevalence (n/%)	Relative abundance (%)		Overall proportion (%)		
			Mean ±Sd	Median (Range)	PRO	CUG	FMT
Anaerostipes	Firmicutes	101/100.0%	3.29±2.54	2.67 (0.41-12.86)	3.92	3.68	1.0
Parabacteroides	Bacteroidetes	100/99.0%	6.63±14.12	0.70 (0.00-50.80)	1.06	0.59	29.5
Anaerotruncus	Firmicutes	95/94.1%	0.22±0.22	0.15 (0.00-1.03)	0.34	0.17	0.0
Oscillibacter	Firmicutes	94/93.1%	1.51±1.73	0.79 (0.00-6.98)	2.93	0.19	0.5
Clostridium XlVb	Firmicutes	90/89.1%	0.21±0.23	0.13 (0.00-1.37)	0.31	0.17	0.0
Flavonifractor	Firmicutes	89/88.1%	0.22±0.28	0.10 (0.00-1.79)	0.21	0.06	0.6
Bacteroides	Bacteroidetes	86/85.1%	2.35±3.95	1.55 (0.00-36.24)	3.61	1.73	0.0
Barnesiella	Bacteroidetes	82/81.2%	4.83±5.23	2.39 (0.00-19.30)	8.79	1.64	0.0
Alistipes	Bacteroidetes	81/80.2%	1.52±1.78	0.87 (0.00-6.54)	2.94	0.32	0.0
Helicobacter	Proteobacteria	81/80.2%	1.21±1.66	0.46 (0.00-9.64)	2.35	0.23	0.0
Saccharibacteria_genera_incertae_sedis	Candidatus Saccharibacteria	81/80.2%	0.40±0.53	0.24（0.00-3.86）	0.55	0.38	0.0
Prevotella	Bacteroidetes	80/79.2%	0.81±1.05	0.50 (0.00-5.85)	0.88	1.13	0.0
Lachnoanaerobaculum	Firmicutes	77/76.2%	0.68±1.03	0.23 (0.00-4.82)	1.33	0.17	0.0
Lactobacillus	Firmicutes	77/76.2%	10.51±16.78	0.32 (0.00-63.30)	0.37	30.62	0.0
Intestinimonas	Firmicutes	76/75.2%	0.30±0.38	0.13 (0.00-1.48)	0.59	0.07	0.0
Roseburia	Firmicutes	76/75.2%	0.20±0.35	0.05 (0.00-2.52)	0.38	0.04	0.0
Alloprevotella	Bacteroidetes	74/73.3%	1.38±2.17	0.32 (0.00-9.91)	2.35	0.56	0.0
Rikenella	Bacteroidetes	74/73.3%	0.15±0.22	0.08 (0.00-1.34)	0.17	0.19	0.0
Enterorhabdus	Actinobacteria	74/73.3%	0.40±0.56	0.11（0.00-2.29）	0.12	0.99	0.0
Erysipelotrichaceae_incertae_sedis	Firmicutes	71/70.3%	0.32±0.52	0.06（0.00-1.95）	0.03	0.27	1.2
Eggerthella	Actinobacteria	70/69.3%	0.07±0.09	0.04（0.00-0.33）	0.02	0.09	0.2

Allobaculum	Firmicutes	65/64.4%	0.94±2.77	0.20（0.00-20.47）	0.71	1.84	0.0
Lachnospiracea_incertae_sedis	Firmicutes	65/64.4%	0.24±0.42	0.09（0.00-2.18）	0.23	0.24	0.2
Pseudoflavonifractor	Firmicutes	64/63.4%	0.15±0.21	0.05（0.00-0.81）	0.31	0.02	0.0
Bifidobacterium	Actinobacteria	63/62.4%	0.54±1.15	0.10（0.00-5.89）	0.43	1.02	0.0
Marvinbryantia	Firmicutes	61/60.4%	0.21±0.30	0.08（0.00-1.44）	0.36	0.1	0.0
Mucispirillum	Deferribacteres	61/60.4%	0.16±0.23	0.06（0.00-1.03）	0.32	0.06	0.0
Clostridium XlVa	Firmicutes	60/59.4%	4.16±8.92	0.05（0.00-30.66）	0.15	0	21.6
Blautia	Firmicutes	60/59.4%	0.87±2.43	0.03（0.00-17.44）	0.08	0	4.5
Anaerofilum	Firmicutes	59/58.4%	0.05±0.06	0.02（0.00-0.27）	0.06	0.01	0.1
Parasutterella	Proteobacteria	58/57.4%	0.15±0.25	0.03（0.00-1.38）	0.09	0.32	0.0
Odoribacter	Bacteroidetes	56/55.4%	0.09±0.18	0.02（0.00-1.09）	0.16	0.02	0.0
Olsenella	Actinobacteria	54/53.5%	0.13±0.29	0.02（0.00-1.90）	0.16	0.16	0.0
Turicibacter	Firmicutes	53/52.5%	1.38±3.67	0.02（0.00-23.07）	0.06	3.87	0.0
Gordonibacter	Actinobacteria	53/52.5%	0.04±0.08	0.02（0.00-0.50）	0.01	0.05	0.1
Ruminococcus	Firmicutes	52/51.5%	0.26±0.74	0.00（0.00-4.94）	0.06	0.04	1.4
Acetatifactor	Firmicutes	51/50.5%	0.19±0.74	0.02（0.00-6.77）	0.14	0.42	0.0
Total 37 healthy core genera			46.77±15.91	42.89（21.00-81.11）	36.60	51.49	60.9

Supplementary Table S3 Numbers of OTUs belonging to the 37 healthy core genera in the three datasets.

Dataset ID	PRO	CUG	FMT
Anaerostipes	12	3	2
Parabacteroides	7	3	3
Anaerotruncus	4	2	1
Oscillibacter	13	1	4
Clostridium XlVb	4	3	1
Flavonifractor	6	3	1
Bacteroides	19	8	4
Barnesiella	16	7	0
Alistipes	11	5	0
Helicobacter	4	1	0
Saccharibacteria_genera_incertae_sedis	1	1	0
Prevotella	5	4	2
Lachnoanaerobaculum	9	3	0
Lactobacillus	5	16	0
Intestinimonas	6	3	1
Roseburia	6	3	2
Alloprevotella	1	1	0
Rikenella	1	2	0
Enterorhabdus	3	2	0
Erysipelotrichaceae_incertae_sedis	2	4	1
Eggerthella	1	2	1
Allobaculum	7	4	0
Lachnospiracea_incertae_sedis	11	1	2
Pseudoflavonifractor	4	1	0
Bifidobacterium	1	2	1
Marvinbryantia	7	2	0
Mucispirillum	1	2	0
Clostridium XlVa	2	0	14
Blautia	4	1	4
Anaerofilum	1	1	1
Parasutterella	2	3	0
Odoribacter	1	1	0
Olsenella	1	2	0
Turicibacter	1	2	0
Gordonibacter	1	2	1
Ruminococcus	3	2	2
Acetatifactor	3	2	0

Supplementary Table S4 The nearest neighbours of the 92 most abundant OTUs belonging to the 37 healthy core genera in the three datasets

OTU	The nearest neighbor	Max score	Total score	Query cover	E value	Identity	Accession
Anaerostipes PRO-OTU01	*Anaerostipes butyraticus*	254	254	100%	1.00E-67	100%	NR_113319.1
Anaerostipes CUG-OTU01	*Anaerostipes butyraticus*	270	270	94%	1.00E-72	99%	NR_113319.1
Anaerostipes FMT-OTU01	*Anaerostipes caccae*	739	739	100%	0	99%	NR_028915.1
Parabacteroides PRO-OTU01	*Parabacteroides merdae*	224	224	100%	1.00E-58	92%	NR_041343.1
Parabacteroides CUG-OTU01	*Parabacteroides merdae*	246	246	95%	2.00E-65	93%	NR_041343.1
Parabacteroides FMT-OTU01	*Parabacteroides merdae*	780	780	100%	0	100%	NR_041343.1
Anaerotruncus PRO-OTU01	*Anaerotruncus rubiinfantis*	243	243	100%	2.00E-64	99%	NR_147398.1
Anaerotruncus CUG-OTU01	*Anaerotruncus rubiinfantis*	272	272	94%	3.00E-73	99%	NR_147398.1
Anaerotruncus FMT-OTU01	*Anaerotruncus colihominis*	741	741	100%	0	100%	NR_027558.1
Oscillibacter PRO-OTU01	*Oscillibacter valericigenes*	235	235	100%	4.00E-62	97%	NR_074793.2
Oscillibacter CUG-OTU01	*Oscillibacter valericigenes*	281	281	94%	6.00E-76	99%	NR_074793.2
Oscillibacter FMT-OTU01	*Oscillibacter ruminantium*	652	652	100%	0	96%	NR_118156.1
Clostridium XlVb PRO-OTU01	*Clostridium propionicum*	254	254	100%	1.00E-67	99%	NR_113408.1
Clostridium XlVb CUG-OTU01	*Clostridium lactatifermentans*	255	255	94%	3.00E-68	96%	NR_025651.1
Clostridium XlVb FMT-OTU01	*Clostridium propionicum*	660	660	100%	0	96%	NR_113408.1
Flavonifractor PRO-OTU01	*Pseudoflavonifractor capillosus*	226	226	100%	2.00E-59	96%	NR_025670.1
Flavonifractor CUG-OTU01	*Flavonifractor plautii*	265	265	94%	6.00E-71	97%	NR_043142.1
Flavonifractor FMT-OTU01	*Flavonifractor plautii*	749	749	100%	0	100%	NR_043142.1
Bacteroides PRO-OTU01	*Bacteroides acidifaciens*	291	291	100%	9.00E-79	100%	NR_112931.1
Bacteroides CUG-OTU01	*Bacteroides acidifaciens*	320	320	95%	1.00E-87	100%	NR_112931.1
Bacteroides FMT-OTU01	*Bacteroides vulgatus*	780	780	100%	0	100%	NR_074515.1
Barnesiella PRO-OTU01	*Muribaculum intestinale*	204	204	100%	1.00E-52	90%	NR_144616.1

Barnesiella CUG-OTU01	*Barnesiella intestinihominis*	204	204	95%	1.00E-52	88%	NR_113073.1
Alistipes PRO-OTU01	*Alistipes senegalensis*	274	274	100%	9.00E-74	98%	NR_118219.1
Alistipes CUG-OTU01	*Alistipes putredinis*	315	315	95%	6.00E-86	99%	NR_113152.1
Helicobacter PRO-OTU01	*Helicobacter valdiviensis*	254	254	100%	1.00E-67	100%	NR_133761.1
Helicobacter CUG-OTU01	*Helicobacter valdiviensis*	283	283	94%	2.00E-76	100%	NR_133761.1
Saccharibacteria_genera_incertae_sedis PRO-OTU01	*Mycoplasma glycophilum*	132	132	69%	5.00E-31	92%	NR_025184.1
Saccharibacteria_genera_incertae_sedis CUG-OTU01	*Mycoplasma glycophilum*	165	165	69%	6.00E-41	93%	NR_025184.1
Prevotella PRO-OTU01	*Prevotella colorans*	235	235	100%	4.00E-62	94%	NR_151886.1
Prevotella CUG-OTU01	*Prevotella colorans*	265	265	95%	6.00E-71	94%	NR_151886.1
Prevotella FMT-OTU01	*Prevotella copri*	749	749	100%	0	99%	NR_040877.1
Lachnoanaerobaculum PRO-OTU01	*Lachnoanaerobaculum umeaense*	239	239	100%	3.00E-63	98%	NR_116814.1
Lachnoanaerobaculum CUG-OTU01	*Lachnoanaerobaculum umeaense*	268	268	84%	4.00E-72	98%	NR_116814.1
Lactobacillus PRO-OTU01	*Lactobacillus animalis*	300	300	100%	2.00E-81	100%	NR_041610.1
Lactobacillus CUG-OTU01	*Lactobacillus johnsonii*	329	329	84%	2.00E-90	100%	NR_117574.1
Intestinimonas PRO-OTU01	*Intestinimonas butyriciproducens*	243	243	100%	3.00E-64	98%	NR_118554.1
Intestinimonas CUG-OTU01	*Intestinimonas butyriciproducens*	272	272	94%	3.00E-73	98%	NR_118554.1
Intestinimonas FMT-OTU01	*Intestinimonas butyriciproducens*	749	749	100%	0	100%	NR_118554.1
Roseburia PRO-OTU01	*Roseburia hominis*	250	250	100%	1.00E-66	99%	NR_074809.1
Roseburia CUG-OTU01	*Roseburia intestinalis*	270	270	94%	1.00E-72	98%	NR_117758.1
Roseburia FMT-OTU01	*Roseburia inulinivorans*	725	725	100%	0	99%	NR_042007.1
Alloprevotella PRO-OTU01	*Prevotellamassilia timonensis*	243	243	100%	2.00E-64	94%	NR_144750.1
Alloprevotella CUG-OTU01	*Prevotellamassilia timonensis*	265	265	95%	6.00E-71	94%	NR_144750.1
Rikenella PRO-OTU01	*Alistipes inops*	202	202	100%	4.00E-52	90%	NR_145882.1
Rikenella CUG-OTU01	*Rikenella microfusus*	257	257	95%	1.00E-68	94%	NR_025910.1
Enterorhabdus PRO-OTU01	*Enterorhabdus caecimuris*	239	239	100%	3.00E-63	98%	NR_115868.1
Enterorhabdus CUG-OTU01	*Enterorhabdus caecimuris*	250	250	94%	2.00E-66	96%	NR_115868.1

Erysipelotrichaceae_incertae_sedis PRO-OTU01	*Eubacterium dolichum*	222	222	100%	3.00E-58	92%	NR_044647.2
Erysipelotrichaceae_incertae_sedis CUG-OTU01	*Eubacterium dolichum*	252	252	95%	4.00E-67	92%	NR_044647.2
Erysipelotrichaceae_incertae_sedis FMT-OTU01	*Clostridium innocuum*	767	767	100%	0	99%	NR_029164.1
Eggerthella PRO-OTU01	*Eggerthella sinensis*	237	237	100%	1.00E-62	98%	NR_042840.1
Eggerthella CUG-OTU01	*Eggerthella sinensis*	267	267	94%	2.00E-71	98%	NR_042840.1
Eggerthella FMT-OTU01	*Eggerthella sinensis*	743	743	100%	0	100%	NR_042840.1
Allobaculum PRO-OTU01	*Allobaculum stercoricanis*	161	161	100%	7.00E-40	85%	NR_042110.1
Allobaculum CUG-OTU01	*Allobaculum stercoricanis*	191	191	95%	1.00E-48	86%	NR_042110.1
Lachnospiracea_incertae_sedis PRO-OTU01	*Eubacterium ventriosum*	244	244	100%	6.00E-65	99%	NR_118670.1
Lachnospiracea_incertae_sedis CUG-OTU01	*Eubacterium ventriosum*	265	265	94%	6.00E-71	98%	NR_118670.1
Lachnospiracea_incertae_sedis FMT-OTU01	*Eubacterium ventriosum*	728	728	100%	0	99%	NR_118670.1
Pseudoflavonifractor PRO-OTU01	*Flintibacter butyricus*	248	248	100%	5.00E-66	99%	NR_144611.1
Pseudoflavonifractor CUG-OTU01	*Intestinimonas butyriciproducens*	237	237	94%	1.00E-62	94%	NR_118554.1
Bifidobacterium PRO-OTU01	*Bifidobacterium pseudolongum*	274	274	100%	8.00E-74	99%	NR_043442.2
Bifidobacterium CUG-OTU01	*Bifidobacterium pseudolongum*	287	287	95%	1.00E-77	97%	NR_043442.2
Bifidobacterium FMT-OTU01	*Bifidobacterium pseudocatenulatum*	756	756	100%	0	100%	NR_037117.1
Marvinbryantia PRO-OTU01	*Marvinbryantia formatexigens*	220	220	100%	1.00E-57	96%	NR_042152.1
Marvinbryantia CUG-OTU01	*Marvinbryantia formatexigens*	206	206	94%	4.00E-53	91%	NR_042152.1
Mucispirillum PRO-OTU01	*Mucispirillum schaedleri*	250	250	100%	1.00E-66	94%	NR_042896.1
Mucispirillum CUG-OTU01	*Mucispirillum schaedleri*	279	279	95%	2.00E-75	95%	NR_042896.1
Clostridium XlVa PRO-OTU01	*Anaerotaenia torta*	237	237	100%	1.00E-62	98%	NR_151894.1
Clostridium XlVa FMT-OTU01	*Clostridium hathewayi*	737	737	100%	0	99%	NR_036928.1
Blautia PRO-OTU01	*Blautia producta*	248	248	100%	5.00E-66	99%	NR_119217.1
Blautia CUG-OTU01	*Blautia marasmi*	261	261	94%	7.00E-70	97%	NR_147395.1
Blautia FMT-OTU01	*Blautia wexlerae*	743	743	100%	0	100%	NR_044054.1
Anaerofilum PRO-OTU01	*Anaerofilum pentosovorans*	219	219	100%	4.00E-57	95%	NR_029313.1

Anaerofilum CUG-OTU01	*Anaerofilum pentosovorans*	244	244	94%	7.00E-65	95%	NR_029313.1
Anaerofilum FMT-OTU01	*Anaerofilum pentosovorans*	686	686	100%	0	97%	NR_029313.1
Parasutterella PRO-OTU01	*Parasutterella excrementihominis*	239	239	100%	3.00E-63	93%	NR_041667.1
Parasutterella CUG-OTU01	*Parasutterella excrementihominis*	261	261	95%	7.00E-70	93%	NR_041667.1
Odoribacter PRO-OTU01	*Odoribacter splanchnicus*	230	230	100%	2.00E-60	93%	NR_074535.1
Odoribacter CUG-OTU01	*Odoribacter splanchnicus*	252	252	95%	4.00E-67	93%	NR_074535.1
Olsenella PRO-OTU01	*Olsenella profusa*	233	233	100%	1.00E-61	96%	NR_116938.1
Olsenella CUG-OTU01	*Olsenella scatoligenes*	255	255	94%	3.00E-68	96%	NR_134781.1
Turicibacter PRO-OTU01	*Turicibacter sanguinis*	239	239	100%	3.00E-63	93%	NR_028816.1
Turicibacter CUG-OTU01	*Turicibacter sanguinis*	268	268	95%	4.00E-72	94%	NR_028816.1
Gordonibacter PRO-OTU01	*Gordonibacter pamelaeae*	220	220	100%	1.00E-57	96%	NR_102934.1
Gordonibacter CUG-OTU01	*Gordonibacter faecihominis*	255	255	94%	3.00E-68	97%	NR_148261.1
Gordonibacter FMT-OTU01	*Gordonibacter pamelaeae*	743	743	100%	0	100%	NR_102934.1
Ruminococcus PRO-OTU01	*Ruminococcus flavefaciens*	209	209	100%	2.00E-54	94%	NR_025931.1
Ruminococcus CUG-OTU01	*Ruminococcus flavefaciens*	283	283	94%	2.00E-76	100%	NR_025931.1
Ruminococcus FMT-OTU01	*Ruminococcus albus*	638	638	100%	0	95%	NR_074399.1
Acetatifactor PRO-OTU01	*Acetatifactor muris*	243	243	100%	2.00E-64	99%	NR_117905.1
Acetatifactor CUG-OTU01	*Acetatifactor muris*	259	259	94%	3.00E-69	97%	NR_117905.1

OTU01: the most abundant OTU in the genus in each dataset.
